# The effects of poliomyelitis on motor unit behavior during repetitive muscle actions: a case report

**DOI:** 10.1186/1756-0500-7-611

**Published:** 2014-09-06

**Authors:** Michael A Trevino, Trent J Herda, Michael A Cooper

**Affiliations:** Neuromechanics Laboratory, Department of Health, Sport, and Exercise Sciences, University of Kansas, 1301 Sunnyside Ave, Room 101BE, Lawrence, KS USA; Dept. of Anatomy and Cell Biology, University of Kansas Medical Center, Kansas City, KS USA

**Keywords:** Electromyography, Firing rates, Isometric, Motorneuron, Vastus lateralis

## Abstract

**Background:**

Acute paralytic poliomyelitis is caused by the poliovirus and usually results in muscle atrophy and weakness occurring in the lower limbs. Indwelling electromyography has been used frequently to investigate the denervation and innervation characteristics of the affected muscle. Recently developed technology allows the decomposition of the raw surface electromyography signals into the firing instances of single motor units. There is limited information regarding this electromyographic decomposition in clinical populations. In addition, regardless of electromyographic methods, no study has examined muscle activation parameters during repetitive muscle actions in polio patients. Therefore, the purpose of this study was to examine the motor unit firing rates and electromyographic amplitude and center frequency of the vastus lateralis during 20 repetitive isometric muscle actions at 50% maximal voluntary contraction in healthy subjects and one patient that acquired acute paralytic poliomyelitis.

**Case presentation:**

One participant that acquired acute type III spinal poliomyelitis (Caucasian male, age = 29 yrs) at 3 months of age and three healthy participants (Caucasian females, age = 19.7 ± 2.1 yrs) participated in this study. The polio participant reported neuromuscular deficiencies as a result of disease in the hips, knees, buttocks, thighs, and lower legs. None of the healthy participants reported any current or ongoing neuromuscular diseases or musculoskeletal injuries.

**Conclusion:**

An acute bout of poliomyelitis altered motor unit behavior, such as, healthy participants displayed greater firing rates than the polio patient. The reduction in motor unit firing rates was likely a fatigue protecting mechanism since denervation via poliomyelitis results in a reduction of motorneurons. In addition, the concurrent changes in motor unit firing rates, electromyography amplitude and frequency for the polio participant would suggest that the entire motorneuron pool was utilized in each contraction unlike for the healthy participants. Finally, healthy participants exhibited changes in all electromyographic parameters during the repetitive muscle actions despite successfully completing all contractions with only a slight reduction in force. Thus, caution is warranted when quantifying muscular fatigue via motor unit firing rates and other electromyographic parameters since the parameters changed despite successful completing of all contractions with only a moderate reduction in strength in healthy subjects.

## Background

Acute paralytic polio is a disease caused by the poliovirus that typically occurs in young children. The poliovirus spreads along nerve pathways and primarily destroys motorneurons of the anterior horns [1]. The poliovirus usually results in asymmetrical paralysis with muscle atrophy and weakness occurring in the lower limbs more so than the upper limbs [2]. Numerous studies have used indwelling electromyography (EMG) to investigate the denervation and reinnervation characteristics of the affected muscle (i.e., motor unit number and amplitude, motor unit firings, conduction velocity, etc.) [3–8]. For example, Larsson *et al.* [7] reported that healthy participants had greater motor unit (MU) firing rates of the tibialis anterior muscle than the poliomyelitis participants during slowly increasing isometric voluntary muscle actions. In addition, McComas *et al.* [9] reported a reduction in motor unit number after an acute bout of poliomyelitis at twice the rate than healthy subjects aged > 60 years, whereas, Herda and Cooper [10] reported a reduction in MUs recruited above 20% of maximal voluntary contraction (MVC) for a poliomyelitis patient in comparison to healthy participants.

Recently, De Luca *et al.* [11] developed technology able to decompose the raw surface EMG signals recorded during isometric muscle actions into its constituent MU action potential trains with the Precision Decomposition (PD) III algorithm. The resulting output allows for the examination of the firing instances for each detected MU. The PD III algorithm has typically detected 30-40 MUs and occasionally up to 60 MUs during a single isometric trapezoid muscle action at various force levels (5 – 100% MVC). Previously, De Luca *et al.* [11] and Nawab *et al.* [12] validated the PD III algorithm and reconstruct-and-test (used for testing accuracy of MU action potential trains) algorithms. This decomposition technology has been used to examine the MU control scheme, which states that lower threshold MUs have higher firing rates than higher threshold MUs during isometric muscle actions [13–15]. However, there is limited information available regarding the use of EMG decomposition in clinical populations [10, 16]. It would be expected that an acute bout of paralytic polio would influence the overall MU control scheme and be detectable by the EMG decomposition technique developed by De Luca *et al.* [11], which was supported by Herda and Cooper [10].

Previous studies have utilized repetitive isometric muscle actions to further elucidate mechanisms of fatigue on MU behavior [17, 18]. For example, Carpentier *et al.* [18] indicated that central drive intensified, as measured by EMG amplitude, while MU discharge rates decreased progressively during the repetitive muscle actions of the first dorsal interosseous at 50% MVC. In addition, the authors reported that the level of change in MU behavior was related to recruitment threshold (low- versus high-threshold), which was also supported by Farina *et al.* [17]. To date, it is unknown the influence of poliomyelitis on MU firing rates and other EMG parameters (amplitude and center frequency) during repetitive muscle actions. Since polio patients have far less motorneurons as a result of the denervation and reinnervation process, it is conceivable that their MU behavior during the repetitive tasks would be altered in comparison to healthy subjects. Therefore, the purpose of this study was to examine the MU control scheme and EMG amplitude and frequency of the vastus lateralis (VL) during 20 repetitive isometric muscle actions at 50% MVC for healthy individuals and one individual that acquired acute paralytic poliomyelitis.

## Case presentation

In this case report, we present one participant that acquired acute type III spinal poliomyelitis (mean ± standard deviation [SD], Caucasian male, age = 29 yrs, weight = 63.4 kg, height = 150.7 cm) at 3 months of age and three healthy participants (Caucasian females, age = 19.7 ± 2.1 yrs, weight = 67.5 ± 9.4 kg, height = 170.7 ± 2.3 cm). Previous research has reported no sex-related differences in EMG amplitude, EMG frequency, or MU behavior during fatiguing contractions [19–25].

The participant with acute type III spinal poliomyelitis (PO) reported neuromuscular deficiencies as a result of disease in the hips, knees, buttocks, thighs, and lower legs. None of the healthy (HE) participants reported any current or ongoing neuromuscular diseases or musculoskeletal injuries. Each participant signed a written informed consent document. This study was approved by the Human Subjects Committee – Lawrence at the University of Kansas.

### Strength testing

Each participant was seated with restraining straps over the pelvis, trunk, and contralateral thigh, and the lateral condyle of the femur was aligned with the input axis of the Biodex System 3 isokinetic dynamometer (Biodex Medical Systems, Inc., Shirley, NY, USA). All isometric leg extensor strength assessments were performed on the right leg at a flexion of 90°. Isometric strength for the leg extensors muscles was measured using the force signal from a load cell (LC402, Omegadyne, Inc., Sunbury, OH, USA) that was fitted to the isokinetic dynamometer.

During the experimental trial, participants completed a warm-up consisting of three to five voluntary isometric muscle actions from 30%-80% MVC. Participants then performed three isometric MVCs with strong verbal encouragement for motivation followed by twenty repetitive submaximal isometric trapezoid muscle actions at 50% MVC. The trapezoid trajectory contained five segments: a 5 sec quiescent period for baseline noise calculation, an up-ramp increased at a rate of 10% MVC/sec, a constant force of 50% MVC for 12 sec, a down-ramp decreased at 10% MVC/sec, and a 3 sec quiescent period. Therefore, the duration of each repetitive trial lasted 22 sec and subjects were given a rest period of 8 – 9 sec between trials. Each participant was instructed to maintain their force output as close as possible to the target force presented digitally in real time on a computer monitor. Following the repetitive muscle actions, participants performed an additional MVC.

### Electromyography signal detection and processing

Surface EMG signals were recorded from the VL using a 5 pin surface array sensor (Delsys, Boston, MA). Prior to sensor placement, the surface of the skin was prepared by shaving, removing superficial dead skin with adhesive tape, and sterilized with an alcohol swab. In addition, EMG signals must successfully pass the Delsys Signal Quality Check (SQC) prior to the recording of EMG for decomposition. The SQC requires the signal to noise ratio to be > 2, baseline noise < 4.8 μV root mean square, and line interference < 1.0 (normalized magnitude power spectrum at 50/60 Hz) (Delsys, Inc., User’s Guide). The surface EMG sensor was placed over the belly of the VL and fixed with adhesive tape while the reference electrode was placed over the patella. Refer to De Luca *et al.* [26] for detailed information regarding the signal processing of the EMG signals. Decomposition techniques (PD III algorithm) were applied to the surface EMG signals to extract action potentials and the firing events of single MUs [11, 12, 14, 27]. Furthermore, the accuracy level of each MU was assessed by a reconstruct-and-test procedure [11]. Only MUs with > 90% accuracy were included for analysis. For each MU, the recruitment thresholds (expressed relative to MVC) and the mean firing rate (MFR) at the targeted contraction level (pulses per second [pps]) were analyzed. The MFR was calculated as the average value of the mean firing rate trajectory during steady force [26]. Linear regressions were performed on the MFR versus recruitment threshold relationships for each muscle action. Slopes and y-intercepts were calculated for each linear regression model. The y-intercept values specify a maximal firing rate (pps) sustainable by the lowest threshold MUs.

In addition, EMG amplitude and mean power frequency (MPF) was investigated. The signals were collected at a frequency of 20 kHz and bandpass filtered (fourth-order Butterworth) at 10-500 Hz. Signal processing was performed with custom programs written with LabVIEW software (Version 11.0, National Instruments, Austin, TX). The amplitude for the EMG signal was calculated with root mean square (RMS). For the isometric muscle actions, EMG RMS and MPF were calculated by averaging the values across the four EMG channels during the entire 12 sec targeted contraction force. These data were normalized to the EMG RMS and MPF values corresponding to the first 50% repetitive muscle action.

## Results

**Figure 1:**
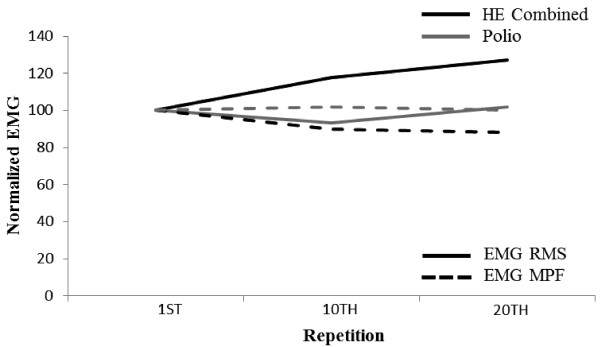
**Plotted normalized electromyography amplitude (EMG RMS) (solid line) and mean power frequency (MPF) (dashed line) values for the healthy (HE) (dark line) and polio (PO) (grey line) participants during the 1st, 10th, and 20th repetitive contraction at 50% maximal voluntary contraction.** From repetition 1 to 20, HE had an increase in RMS (26.97%) and a decrease in MPF (11.81%) while the PO had relatively no change in RMS (1.71%) or MPF (0%).

**Figure 2:**
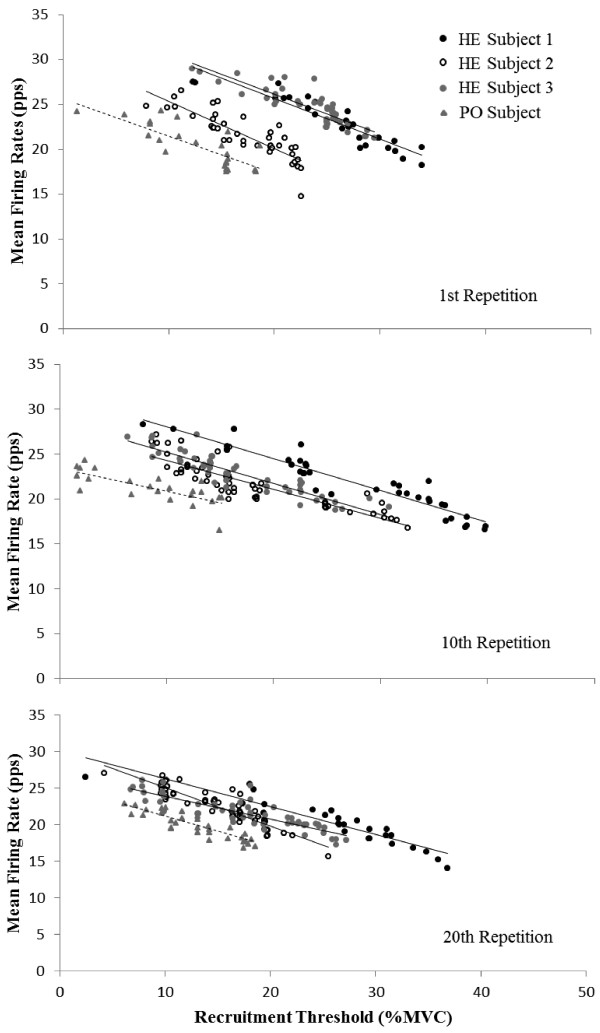
**Plotted mean firing rate (MFR) versus recruitment threshold (expressed relative to% maximal voluntary contraction [MVC]) relationships from the VL for the healthy (HE) (subject 1 – black circular markers, subject 2 – empty black circular markers, subject 3 – grey circular markers; solid linear regression lines) and polio (PO) participants (grey triangular markers; dashed regression line) during the 1st, 10th, and 20th repetitive contraction at 50% MVC.** From repetition 1 to 20, HE experienced a greater decrease in y-intercepts (12.86%) than PO (1.50%).

There was a total of 383 MUs analyzed for the HE participants with 80 MUs analyzed for the PO participant (Table 1). For the pre- and post-MVCs, peak force values decreased 10.3% and 28.0% for the HE (548.67 ± 121.01 N, 492.00 ± 147.15 N) and PO (37.80 N, 27.20 N), respectively. The normalized (i.e., 1^st^ repetition = 100%) EMG RMS was 117.68 ± 9.36% and 126.97 ± 23.13% while the MPF was 89.90 ± 1.90% and 88.19 ± 4.06% for the HE during the 10^th^ and 20^th^ repetition, respectively (Figure 1). For the PO, the normalized EMG RMS was 93.37% and 101.71% while the MPF was 101.78% and 100.00% during the 10^th^ and 20^th^ repetitive contraction (Figure 1). For the y-intercepts, the mean ± SD for the HE were 33.42 ± 2.41 pps, 29.27 ± 2.12 pps, and 29.12 ± 1.83 pps for the 1^st^, 10^th^, and 20^th^ repetition and the y-intercepts for the PO were 25.64 pps, 23.48 pps, and 25.26 pps for the 1^st^, 10^th^, and 20^th^ repetition (Figure 2). For the slopes, the mean ± SD for the HE were -0.47 ± 0.05, -0.34 ± 0.02, and -0.41 ± 0.11 for the 1^st^, 10^th^, and 20^th^ repetition and the slopes for the PO for the 1^st^, 10^th^, and 20^th^ repetition were -0.42, -0.26, and -0.41 (Figure 2).

**Table 1:** **Motor unit (MU) number, slope, y-intercept and R values for the healthy (HE) and acute paralytic poliomyelitis (PO) participants and repetitive (Rep) contractions (1st, 10th, and 20th) for the mean firing rate (MFR) versus recruitment threshold**

Subject	MUs	Rep#	MFR vs recruitment threshold		
			Slope	Y-intercept	R
1HE	28	**1st**	-0.452	34.697	0.925
	36	**10th**	-0.356	31.632	0.947
	26	**20th**	-0.381	30.078	0.922
2HE	41	**1st**	-0.526	30.641	0.858
	55	**10th**	-0.323	27.535	0.874
	51	**20th**	-0.523	30.283	0.914
3HE	43	**1st**	-0.439	34.926	0.862
	50	**10th**	-0.345	28.641	0.909
	53	**20th**	-0.313	27.011	0.866
1PO	24	**1st**	-0.418	25.642	0.798
	23	**10th**	-0.261	23.478	0.755
	33	**20th**	-0.412	25.257	0.836

All relationships were significant (P < 0.05).

## Discussion

Previously, Larsson *et al.* [7] reported differences in MU firing rates between healthy and prior polio patients during “*slowly increasing isometric voluntary contractions*”^(p.190)^. The y-intercept from the MFR versus recruitment threshold relationships theoretically represents a maximal firing rate sustainable by the lowest threshold MUs [14]. In the present study, the MFRs for the HE were higher (y-intercept range: 33.42 ± 2.41 – 29.12 ± 1.83 pps) than the PO (y-intercept range: 25.64 - 23.48 pps) for each repetition (Figure 2). Negative slopes were reported from the linear regressions for each participant and contraction and, thus, earlier recruited MUs tended to have greater firing rates than the later recruited MUs. Referring to Table 1 and Figure 2, there were no observed differences between the slopes between PO and HE participants. Therefore, the inverse relationship between the firing rate and recruitment threshold typically reported in healthy muscle was present for the PO muscle [14]. The overall MU control scheme (i.e., negative slopes) was similar between the PO and HE participants, however, the MFRs were less (i.e., y-intercepts) for the PO than the HE participants. High-threshold MUs are typically described as fast fatiguing for the VL. De Luca and Hostage [14] suggested that if the high-threshold MUs discharged at relatively higher rates, high-threshold MUs would fatigue more quickly and force will not be maintained. Since it has been reported that there are far less motorneurons in the PO than HE participants as a result of denervation [5, 8], the lower MFRs throughout the force spectrum for the PO is possibly a fatigue protecting mechanism.

EMG RMS is a global measure of MU recruitment and firing rates while EMG MPF reflects the average muscle fiber conduction velocity [28]. Classically, muscle fatigue has been monitored with these EMG parameters and indicators of fatigue are believed to be evident by increases in RMS and decreases in MPF [29]. An increase in EMG RMS is the result of synchronization of active MUs and the recruitment of new MUs while the decrease in MPF has been primarily attributed to a reduction in muscle fiber conduction velocity [30]. For the 1^st^ to 10^th^ repetition, the HE had an increase (17.7%) in EMG RMS while EMG MPF declined (10.0%). Concurrently, the y-intercepts from the MFR versus recruitment threshold relationships decreased (12.4%) for the HE participants and, thus, would suggest that the HE participants recruited additional MUs and/or had greater MU synchronization during the 10^th^ repetition in comparison to the 1^st^ repetition. In contrast, the y-intercepts from the MFR versus recruitment threshold relationships and EMG RMS decreased (8.4% and 6.6%) simultaneously while EMG MPF (+1.8%) remained stable from the 1^st^ to 10^th^ repetition for the PO participant. For the 10^th^ to 20^th^ repetition, there was relatively no change in the y-intercepts (0.5%) or EMG MPF (-1.7%), however, EMG RMS increased 9.3% for the HE. For the PO, the y-intercept from the MFR versus recruitment threshold and EMG RMS returned to baseline levels (i.e., increased) during the 20^th^ repetition. The EMG parameters indicated that MU behavior differed between the PO and HE during the repetitive muscle actions. Subsequently, MU firing rates and EMG RMS responded in a similar manner for the PO, in contrast, MU firing rates and EMG MPF decreased while EMG RMS increased during the repetitive muscle actions for the HE. Future studies may want to consider normalizing these parameters to an MVC rather than a submaximal muscle action as in the present study to further elucidate neural mechanisms of fatigue.

Previously, Larsson *et al.* [7] reported that polio patients used muscle fibers in an all or none manner during slowly increasing isometric voluntary muscle actions. In the present study, the changes in EMG RMS mirrored the changes in the y-intercepts from the MFR versus recruitment threshold relationships with relatively no change in MPF for the PO, which tentatively suggested that all MUs were recruited during each muscle action at 50% MVC. Since EMG RMS is the concurrent representation of MU recruitment and firing rates, the increase in EMG RMS with a corresponding decrease in MFR would suggest that HE relied on recruitment of additional MUs and/or synchronization of MU during the repetitive tasks. However, the mirrored changes between EMG RMS and MFR for the PO suggested that no additional MUs were recruited nor was there an increase in MU synchronization during the repetitive tasks. Interestingly, HE participants were able to complete all 20 contractions with a moderate (10.3%) reduction in post-MVC strength. Although the PO had fewer changes in all parameters measured (MU MFR, EMG RMS and MPF), the decrease in post MVC for the PO was much greater than the HE (28.0% vs. 10.3%). Thus, EMG parameters changed during the repetitive tasks despite a lack of fatigue in the HE as indicated by the successful completion of the tasks with only a moderate reduction in strength.

## Conclusions

In summary, the denervation and reinnervation process as a result of an acute bout of poliomyelitis altered MU behavior in comparison to the HE participants. Specifically, the PO participants had lower MFRs and the y-intercepts from the MFR versus recruitment threshold relationships mirrored changes in EMG RMS from the 1^st^ to 20^th^ repetition, unlike the HE participants. Thus, suggesting that all MUs were recruited during each contraction for the PO participant. In addition, caution is warranted when examining EMG RMS and MPF and MU MFR behavior as indicators of muscular fatigue during repetitive muscle actions since the parameters changed in the HE despite successfully completing all muscle actions with only a moderate decrease in strength. Future research should examine the influence of high-intensity exercise training on the fatigability of polio patients as it is evident that the alterations in MU control scheme provide a mechanism to respond to such training despite the denervation and reinnervation process.

## Consent

Written informed consent was obtained from all participants for publication of this Case Report. A copy of the written consent is available for review by the Editor-in-Chief of this journal.

## Abbreviations

EMG: Electromyography
HE: Healthy participant
MFR: Mean firing rate
MPF: Mean power frequency
MU: Motor Unit
MVC: Maximal voluntary contraction
PO: Polio participant
PD: Precision decomposition
PPS: Pulses per second
RMS: Root mean square
SQC: Signal quality check
SD: Standard deviation
VL: Vastus lateralis.
